# A Scheduling Mechanism Based on Optimization Using IoT-Tasks Orchestration for Efficient Patient Health Monitoring

**DOI:** 10.3390/s21165430

**Published:** 2021-08-11

**Authors:** Naeem Iqbal, Imran, Shabir Ahmad, Rashid Ahmad, Do-Hyeun Kim

**Affiliations:** 1 Department of Computer Engineering, Jeju National University, Jeju 63243, Korea; naeemiqbal@jejunu.ac.kr (N.I.); imranjofficial@jejunu.ac.kr (I.); 2 Department of IT Convergence Engineering, Gachon University, Seongnam 13120, Korea; shabir@gachon.ac.kr; 3 Department of Computer Science, COMSATS University Islamabad at Attock, Attock 43600, Pakistan; rashid.ahmad@ciit-attock.edu.pk

**Keywords:** Internet of Things, smart healthcare, remote health monitoring, vital signs monitoring, optimization

## Abstract

Over the past years, numerous Internet of Things (IoT)-based healthcare systems have been developed to monitor patient health conditions, but these traditional systems do not adapt to constraints imposed by revolutionized IoT technology. IoT-based healthcare systems are considered mission-critical applications whose missing deadlines cause critical situations. For example, in patients with chronic diseases or other fatal diseases, a missed task could lead to fatalities. This study presents a smart patient health monitoring system (PHMS) based on an optimized scheduling mechanism using IoT-tasks orchestration architecture to monitor vital signs data of remote patients. The proposed smart PHMS consists of two core modules: a healthcare task scheduling based on optimization and optimization of healthcare services using a real-time IoT-based task orchestration architecture. First, an optimized time-constraint-aware scheduling mechanism using a real-time IoT-based task orchestration architecture is developed to generate autonomous healthcare tasks and effectively handle the deployment of emergent healthcare tasks. Second, an optimization module is developed to optimize the services of the e-Health industry based on objective functions. Furthermore, our study uses Libelium e-Health toolkit to monitors the physiological data of remote patients continuously. The experimental results reveal that an optimized scheduling mechanism reduces the tasks starvation by 14% and tasks failure by 17% compared to a conventional fair emergency first (FEF) scheduling mechanism. The performance analysis results demonstrate the effectiveness of the proposed system, and it suggests that the proposed solution can be an effective and sustainable solution towards monitoring patient’s vital signs data in the IoT-based e-Health domain.

## 1. Introduction

Recently, Internet of things (IoT) has been defined as a revolutionary technology to develop tremendous IoT-based applications in various domains, such as smart healthcare, smart home, and smart city [1,2,3]. IoT is a huge network of connected devices, things, and objects, all of which collect and share data about how they are used and the environment around them. The main objective of the IoT platform is to allow humans and computers to interact and communicate with billions of things and objects [4]. IoT platforms for the healthcare domain are more comprehensive than traditional platforms, which use to provide better quality services to the healthcare industry [5]. IoT devices for the smart healthcare domain such as body and environmental sensors, actuators, and motion sensors work at the lowest layer. These connected devices are linked with devices of the communication layer, which use to collect sensing data from IoT devices and transfer it over the internet for further processing to address specific needs. Doctors and healthcare researchers carefully analyze the acquired sensing data using statistical analysis applications. Over the past years, various IoT-based healthcare applications have been developed to collect, process, and analyze vital signs data acquired from sensors equipped with patient’s body [6,7,8,9,10,11].

Today, electronic health (e-Health) is an emerging health service paradigm that uses information communication technologies (ICTs) to enhance traditional healthcare systems to provide better health services to remote patients. These systems are used to monitor the vital signs data of remote patients, which is transferred to the doctor through communication devices and processed for diagnosis. For instance, chronic disease patients are monitored regularly, such as patients with cardiac disease are monitored by sensing data of electrocardiogram (ECG), and patients with diabetes are monitored by sugar level. According to a study [12], half of the American adult population are diagnosed with at least one chronic condition, and one of three adults are suffering from two or more chronic diseases. Chronic diseases are causes of mortality. Out of 10 mortalities, 10 percent are caused due chronic diseases. Heart and cancer diseases are considered leading causes of death, which is reported 48% of total deaths. In traditional healthcare systems, a large number of healthcare resources, such as doctors, nurses, and therapists, continuously monitor the vital signs of patients. In recent years, different health monitoring systems have been developed and employed to collect and process vital signs data of patients using IoT devices [13,14,15]. IoT-based healthcare applications are more comprehensive to develop quality of service (QoS) requirements that differentiate them from other applications based on IoT technology.

However, there are different features such as delay sensitivity, time criticality, network cost efficiency, fault tolerance, collection, and processing of sensing data that are considered critical for real-time smart health applications based on different reasons [16]. For instance, vital signs data of patients are collected using body sensors that operate on different priorities level to monitor patient’s health conditions. In the medical domain, some of the patient’s vital signs data are considered critical [17]. Traditional healthcare systems are not well-structured to automatically generate healthcare tasks and handle the task allocation process efficiently and dynamically for high-priority healthcare tasks. Therefore, it is required to order healthcare tasks (including priority and event tasks) based on a time-constraint scheduling mechanism to minimize task allocation delays and increase throughput in terms of response time. It is also required to generate a new task in the case of emergent conditions to send notification alerts to concerned authorities in order to take appropriate actions.

Furthermore, safety is considered an essential element in smart e-Health systems. An e-Health service provider is said to be safe if it can self-diagnose sensors faults to provide reliable healthcare services to remote patients. On the other hand, the failure frequencies of sensors are also considered a challenging issue in the smart e-Health domain. If the failure frequency of sensing devices is high, then it causes to degrade the reliability of the smart e-Health system. Therefore, it is required to optimize information lost during context switching from a faulty sensor to a backup sensor and minimize sensors failure frequency to increase the reliability of the smart e-Health system.

In this study, a smart PHMS is developed to monitor the vital signs data of remote patients in the home as well as in the hospital and optimize healthcare services for providing reliable health services to remote patients. Our proposed smart PHMS consists of two main modules; healthcare tasks data monitoring using a real-time IoT-based tasks orchestration architecture and optimization of healthcare services. First, a real-time IoT-based task orchestration architecture is developed based on a self-management paradigm to monitor patient’s vital signs data effectively. Second, an optimization module is formulated using an optimization scheme based on objective function to provide reliable services to remote patients. The self-management task management architecture consists of the following steps; analysis and decomposition of complex problems into micro-problems, generation of healthcare tasks, healthcare tasks mapping, healthcare tasks scheduling using an optimized time-constraint aware scheduling, and deployment of healthcare tasks on physical devices. An optimized PSO-enabled time constraints-aware scheduling mechanism is developed to schedule healthcare tasks to handle emergent tasks effectively. The optimization module aims to optimize healthcare services based on objective function using a meta-heuristic technique to provide a reliable treatment to remote patients. The main objective of task orchestration architecture is to automatically generate healthcare tasks and dynamically handle the deployment of emergent tasks to tackle patient’s critical conditions effectively. The proposed smart PHMS is developed based on a self-management paradigm using web technology, such as front-end technology (CSS3, HTML5, and JavaScript) and backend technology (Python and Flask framework). Furthermore, for experiment purposes, a well-known Libelium e-health toolkit is used to monitor the following vital signs data of patients, such as body temperature, ECG, blood pressure, SpO2, and respiration rate. Moreover, the JMeter tool is used to record simulated data of virtual patients to conduct a series of experiments. Different performance analyses are used to evaluate the significance of the proposed smart PHMS, such as round trip time (RTT), latency, starvation, and drop rates of healthcare tasks.

The notable contributions of the proposed study is followed as:

- Development of a scheduling mechanism based on optimization and time-constraint-aware scheduling techniques using a real-time IoT-based task orchestration architecture to efficiently monitor vital signs data of remote patients;
- Development of an optimization module based on objective function to minimize information lost during context switching of sensing devices and minimize the sensor’s failure rate to improve recovery and reliability of the proposed smart PHMS;
- Development of a PSO-enabled Time-constraint aware scheduling mechanism using a real-time IoT-based task orchestration architecture to schedule healthcare tasks efficiently;
- Development of real-time IoT-based task orchestration architecture to handle the deployment of critical and emergent healthcare tasks effectively and dynamically;
- Utilization of different evaluation metrics are to evaluate the effectiveness of the proposed smart PHMS, such as RTT, latency, throughput, response time, and task drop and starvation rates.

The rest of the paper is divided into the following sections. Section 2 presents the related works; Section 3 presents architecture of the proposed smart PHMS, optimization functionality to overcome recovery and reliability challenges in smart e-Health domain, and  a tasks orchestration mechanism. Section 4 presents experimental and implementation environment of the proposed smart PHMS. Section 5 discusses experimental results, performance analysis and comparison of the proposed system with state-of-art-techniques. Section 6 concludes the paper with possible future direction.

## 2. Related Work

This section presents existing studies related to remote patient health monitoring systems. Advancement in IoT technologies paved a revolution in e-Health systems to provide healthcare services to remote patients. Remote healthcare monitoring is today’s most vital protection system for patients who choose to stay at home for various reasons. Previously the doctor needs to check the patient in the form of physical examinations, and continuous monitoring was impossible. Hence in case of critical emergencies, continuous monitoring was impossible, which could be stressful for both patient and the doctor. In recent years, there are various IoT-based healthcare applications developed to facilitate remote patients in the healthcare domain [18,19,20,21,22]. All these IoT-based healthcare applications are used to monitor, process, and analyze the vital signs data of patients. However, there are different issues faced by traditional e-Health systems, for instance, high delay sensitivity, fault tolerance, network cost efficiency, to name a few. These systems are not consistent to handle tasks allocation process automatically to deploy healthcare tasks efficiently.

In [18], the authors presented an architecture for detection of symptoms associated with heart failure patients comprising of sensors, web servers and databases. This wireless sensor-based system detected symptoms related to heart disease to a greater extent. Jara et al. [19] utilized the sensing abilities of IoT devices and presented a mobile health framework to handle the emergency condition of patients. Another study presented in [23] to utilized smartphones for monitoring patient health status. In [20], the authors presented a detailed review on methods and techniques applied to the domain of health care services for data analysis from various wearable sensors. In [21,24], the authors developed a system for the detection of critical heart-related symptoms based on advanced remote monitoring. T. Klingeber et al. [22] presented an algorithm to improve the data fusion process to record large patient vital signs data precisely, such as ECG, blood pressure, skin temperature, to name of a few. In [25], the authors used a wireless sensor network (WSN) to monitor patient vital signs data. Another study presented in [26] integrated IoT technology in the health monitoring system to monitor and observe the health condition of remote elderly patients. In [27], the authors proposed a multi-parameters based fall detection model for elderly patients. An integrated model was proposed to combine smart-watch and accelerometer to recognize falls of elderly patients [28]. There are different existing studies attempted to utilize accelerometer, gyroscopes, and barometers to detect falls to improve the efficiency of IoT-based health monitoring systems [29,30]. The authors presented a WAN-based energy-efficient system for health care data analysis [31]. Furthermore, both [32,33] proposed two-fold encryption model to secure sensing data of remote patients to ensure data privacy and security.

Different researchers have proposed and developed cloud-based health monitoring systems to facilitate remote patients. In [34], the authors suggested an efficient health care monitoring system for elderly patients based on fog and cloud computing. The novel fog to cloud-based architecture facilitated the management of health data efficiently. In [35], the authors developed a reliable cloud-based system for ECG monitoring; the data is collected and transmitted to the IoT cloud through a wearable monitoring node. Another study presented in [36] suggested a cloud-based architecture to detect and monitor patients having Parkinson’s disease in developing countries. The developed architecture enabled healthcare practitioners to analyze voice samples of patients collected through their phones for detecting and diagnosing Parkinson’s disease. Experimental results demonstrated that the proposed architecture achieved an accuracy of 96.6%. Likewise, to detect heart rate variations, the authors presented an automated cloud-based system [37]. The system involves two databases: MIT Physionet database, and the second was composed of gathering data from thirty people through wearable sensors. In [38], the authors presented a Body-Cloud-based system involving body sensor networks for real-time monitoring of cardiac data.

Health monitoring systems are revolutionized by fog computing architecture, bringing significant improvements in telehealth and medicine. In literature, fog computing-based health monitoring architectures were proposed to address the problems of elderly patients suffering from chronic diseases. These architectures provide efficient network resource utilization, such as network bandwidth, etc. Hence, smart mobile device users are provided with real-time information close to the network edge. Based on these concepts, we can categorize the literature into two sections: fog computing-based patient health care monitoring system and remote health care monitoring system using IoT. In [39], the authors proposed an improved fog computing system based on the cloud. This system aids the real-time applications through analysis of biosignals at the fog server end. In [40], the authors implemented a gateway named smart e-health to be utilized in fog computing layers. A practical implementation of IoT-based early warning score related to health monitoring was also done to prove the efficient working of the system [41].

In IoT-based health applications, task scheduling is an essential process to order healthcare tasks correctly for efficient deployment. The dynamic task scheduling process helps to minimize latency and maximize throughput. However, traditional e-Health systems are developed based on static task generation and scheduling mechanisms to handle the health task allocation process [42]. The authors defined a task allocation problem as an integrated linear model, which aims to minimize latency during resources requested by a particular task. In [43], the authors proposed an event triggering-based model to process real-time data of patients. The authors defined various health tasks to acquire and process real-time patient data. In another study presented in [44], a prototype-based architecture was presented to consider speech motor disorders patient’s data as a case study. The authors focused on the static allocation of health tasks to acquire patients data to analyze and diagnosis for better treatment. In [45], the authors proposed a fall-detection algorithm to detect the fall of elderly patients based on a static task allocation mechanism.

Different researchers propose different task scheduling mechanisms to tackle the allocation process of tasks in different domains [46,47,48,49,50,51]. In [46], the authors developed an efficient IoT-based service delegation and resource allocator system for managing and delegating requests of users to their appropriate fog/cloud. The study presented in [47] utilized predict earliest finish time (PEFT) algorithm that works on the concept of optimistic cost table to prioritize tasks and selecting processor. In [48], the authors proposed a novel method of the clustering algorithm to schedule multi workflows in a cloud computing environment. In [49], the authors proposed a novel solution to the problem of fault tolerance, optimal resource allocation, and minimizing overflow of a resource based on an efficient resource allocator (ERA). In [50], a new scheduling strategy was developed to achieve a trade-off between the performance of the application (in terms of execution) and the associated cost of cloud resource usage to provide better quality services. In [51], the authors used data mining (using Apriori) techniques for task scheduling in Fog computing-based devices. Experimental findings state that the proposed algorithm has achieved better execution time and average wait time. Furthermore, Table 1 summarizes existing health monitoring systems based on different paradigms to facilitate remote patients in homes and hospitals.

To the best of the author’s knowledge, all of the aforementioned IoT-based e-Health systems are developed based on static task scheduling and allocation. Moreover, all these existing are not well-structured to generate autonomous health tasks and handle the task allocation process for high priority health tasks dynamically. Furthermore, all these existing IoT-based e-Health systems faced different issues, such as delay sensitivity, time criticality, high fault tolerance, high network cost, to name a few. Therefore, a new solution is required to monitor patient health conditions based on efficient task orchestration architecture, which aims to minimize latency, maximize throughput, and handle emergent health tasks effectively. It is also required to optimize wireless network cost to drive optimal healthcare tasks scheduling decisions to provide cost-effective health services to remote patients. Furthermore, an ideal IoT-based smart PHMS should provide safety in terms of recovery and reliability.

## 3. Materials and Methods

This section presents materials and methods used for development of the proposed smart PHMS.

### 3.1. Design of Proposed Smart PHMS

**Figure 1:**
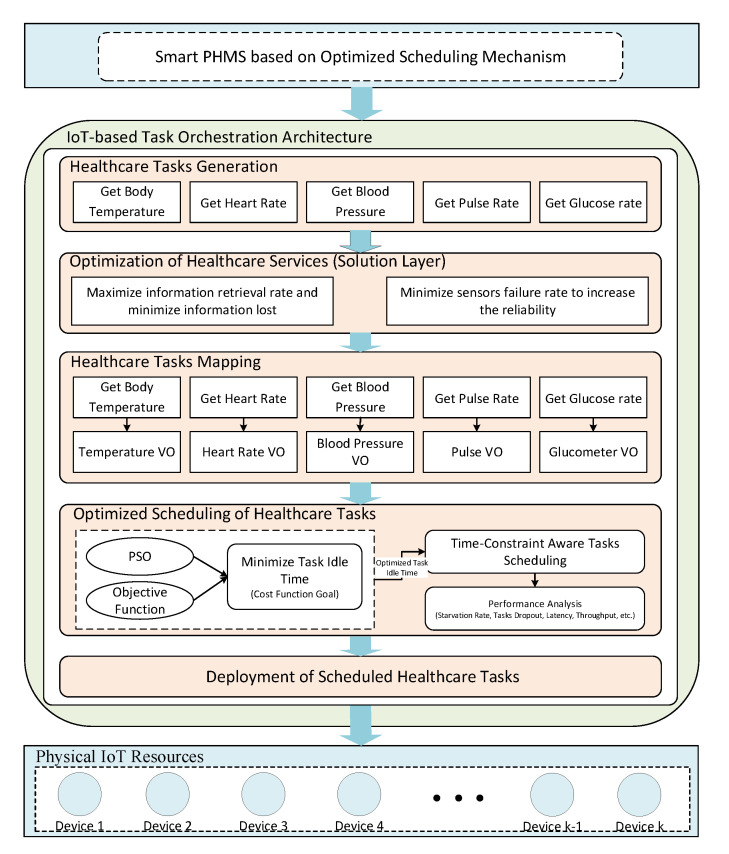
Proposed architecture of Smart patient health monitoring systems (PHMS).

This subsection presents proposed architecture of smart PHMS. Figure 1 presents a proposed architecture diagram of the proposed smart PHMS. The proposed smart PHMS architecture consists of the following layers: generation of healthcare tasks, optimization of healthcare services (solution layer), mapping healthcare tasks on virtual objects, optimized scheduling mechanism, deployment of healthcare tasks on physical IoT devices.

Task generation layer incorporates into following sub-layer to generate healthcare tasks. It includes the decomposition of the problem layer into multiple sub-problems. Each sub-problem is decomposed into healthcare tasks using natural language processing (NLP). In the solution layer, each sub-problem consists of one or many goals, such as proposed smart PHMS is said to be safe if it minimizes information lost during context switching process and sensors failure rate to increase the reliability of the developed system. The solution layer consists of the following mathematical formulations, which aim to minimize information lost during context switching of sensors and minimize sensors failure rate to provide a safe and reliable environment to remote patients. In the healthcare tasks mapping, generated healthcare tasks are mapped on virtual objects (VOs) to formulate tasks and VOs pairs. Next, an optimized task constraint-aware scheduling mechanism is developed based on objective function to optimize task idle time to enhance the scheduling process. In the virtualization layer, virtual objects are generated for physical devices to perform tasks related to the IoT environment. Finally, the physical resource layer consists of the physical IoT devices, such as sensors and actuators nodes. The sensing nodes are used to sense data from installed or equipped sensors with the patient body, while actuators are responsible for executing generated control commands through installed actuators. The ultimate goal of this work is to facilitate remote patients by providing a safe and reliable environment.

### 3.2. Mathematical Formulations for Smart PHMS

This subsection presents mathematical problem formulation to optimize health monitoring services to remote patients. The optimization module aims to minimize information lost during context switching of sensors and tasks failure frequency. The safety problem for remote patient’s health monitoring in both home and hospital is to self-diagnosis sensor fault to increase the reliability of health services for remote patients. This paper uses an optimization approach to formulate information loss recovery (ILR) to minimize information lost during context switching from a faulty sensor and backup sensor. Table 2 presents notations and symbols used in this problem formulation.

There are several assumptions to formulate objective function, for example, *N* number of available sensors to collect data from patients $S_{N}$, *K* number of additional sensors to tackle sensors replacement issues $S_{K}$. Thus, $R(S_{N},S_{K})$ ratio can be calculated as follows in Equation (1).

(1) $$R\left(S_{N},S_{K}\right)=\frac{S_{N}}{S_{K}}$$

ILR index is defined as the ratio of total retrieve information from each sensing device fault $I_{R}$ and the lost information for sensing device fault $I_{L}$. Thus, the $R(I_{R},I_{L})$ ratio is calculated as follows in Equation (2).

(2) $$R\left(I_{R},I_{L}\right)=\frac{\sum I_{R}}{\sum I_{L}}=\frac{I_{R,1}+I_{R,2}+I_{R,3}+\ldots +I_{R,M}}{I_{L,1}+I_{L,2}+I_{L,3}+\ldots +I_{L,M}}$$

By integrating Equation (1) and Equation (2), we form an objective function, which aims to maximize information retrieval rate and minimize information lost during context switching from a faulty sensor to a backup sensor. The following Equation (3) is used to calculate the information loss recovery ILR index:

(3) $$ILR=\frac{R(S_{N},S_{K})}{R(I_{R},I_{L})}=\frac{S_{N}\sum I_{L}}{S_{K}\sum I_{R}}$$

Thus, an objective function is defined as an Equation (4) to minimize ILR index during context switching of sensing devices.

(4) $$ILR=Minimize(\frac{S_{N}\sum I_{L}}{S_{K}\sum I_{R}})$$

The second problem is related to reliability is the failure frequencies of sensors. If the failure frequency is high, then the overall reliability of the system will be low. Therefore, we propose an optimized approach based on the objective function, which aims to minimize sensor’s failure frequency to increase the reliability of the sensing data of the proposed smart PHMS. It enables smart PHMS to verify that acquired sensing data are correct. It validates sensing data of each sensor by using upper and lower threshold values. If sensing values lie between upper and lower threshold values bounds, it ensures that acquired data is corrected; otherwise, it will be considered a sensor fault, and sensing data will not be considered for patient health analysis. There are following assumptions, such that the $i^{th}$ sensor of the patient health monitoring system collects *n* data samples in the time interval *t*, sequence of the data can be represented as $x=x_{1},x_{2},x_{3},\ldots ,x_{n}$, then the real data condition is defined as follows in Equation (5):

(5) $$T_{1}\left(t\right)<x_{n}<T_{2}\left(t\right)$$

where $T_{1}\left(t\right)$ and $T_{2}\left(t\right)$ are threshold functions, such as lower and upper threshold functions of $i^{th}$ sensor at time interval *t*. Both threshold functions validate sensing data whether it lies in a valid range or not. In this way, It will enhance the reliability of the proposed smart PHMS if sensing values fall between lower and upper threshold functions. The performance of distortion fault of sensor discrete point data is defined based on the upper and lower threshold formula shown in Equation (6):

(6) $$x_{n}>T_{1}\left(t\right),\;or\;\;x_{n}<\;T_{2}\left(t\right)$$

The mathematical formulation for sensor distortion fault is followed in Equation (7), where *a* indicates the average value of the ratio of maximum and minimum values of measurement signal data of $i^{th}$ sensor of proposed smart PHMS. The following Equation (7) is used to obtain sensor failure faults for sensing data *x* for $i^{th}$ sensor at time interval *t*.

(7) $$a\left(max,min\right)=\frac{\left(max(x)+min(x)\right)}{2}=\frac{max\left(x_{1},x_{2},\ldots ,x_{n}\right)+min\left(x_{1},x_{2},\ldots ,x_{n}\right)}{2}$$

The linear minimum mean variance of the corresponding fault signal μ is estimated to determine the threshold value of generalized quasi natural ratio [52] is shown in Equation (8).

(8) $$S_{f}=\left(\frac{p(\mu |v;H_{i})}{p(\mu |v;H_{j})}\right)$$

Our objective function aims to minimize sensors failure rate is shown in Equation (9):

(9) $$S_{f}=Minimize\left(\frac{p(\mu |v;H_{i})}{p(\mu |v;H_{j})}\right)$$

The final objective function is defined in Equation (10), which aims to minimize information lost during context switching process of faulty and backup sensors and minimize sensors failure to increase the reliability of the proposed smart PHMS. The α and β are the weighting parameters of the functions ILR and $S_{f}$.

(10) $$S_{index}=Minimize\left(\alpha ILR+\beta S_{f}\right)$$

### 3.3. Task Orchestration Architecture for Smart PHMS

This section presents a detailed architecture of the task orchestration in a health monitoring system, which aims to get sensing data from remote patients in an effective way.

**Figure 2:**
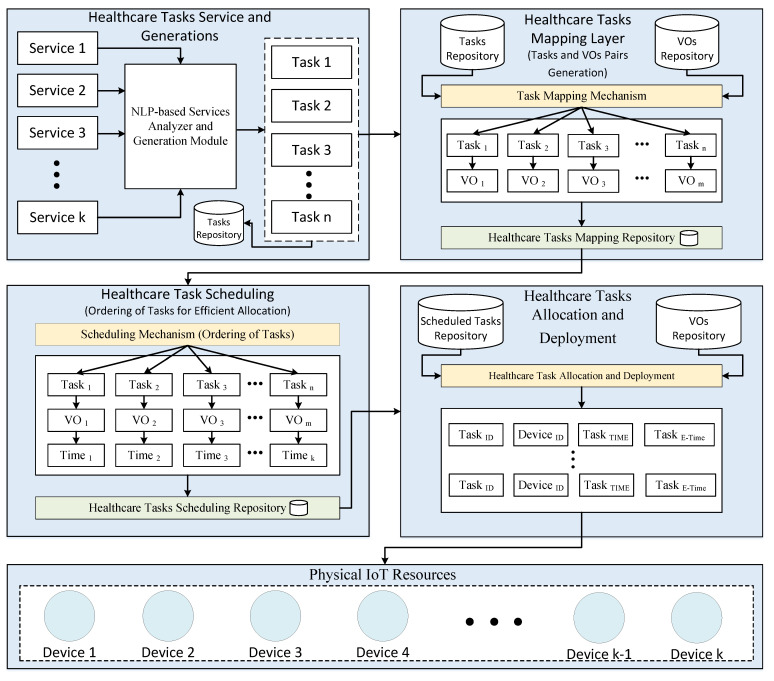
Task Orchestration Architecture for Efficient Tasks Allocation in Smart PHMS.

The proposed task orchestration module consists of the five-layered architecture, such as service analysis and decomposition of services into microservices, task generation using NLP techniques, task/virtual object pair generation and mapping, optimal task scheduling, task allocation and deployment, as shown in Figure 2.

Each layer performs a unique functionality towards the main functional goal. The main objective of the proposed architecture is to utilize task composition through NLP techniques and VOs mechanism to allocate the given health-related tasks on VOs in a smart health monitoring system efficiently. The physical IoT resource layer consists of installed physical devices, such as sensors and actuators. The installed sensors are used to sense and transfer remote patient’s health data to IoT servers. There are different sensors equipped with the patient body, for instance, temperature sensor, heart rate sensor, blood pressure sensor, pulse rate sensor, to name a few. In addition, the following actuators, such as alarm and notification actuators, are used to send alert messages to healthcare practitioners in an emergency.

#### 3.3.1. Service Analysis and Decomposition of Health Tasks

**Figure 3:**
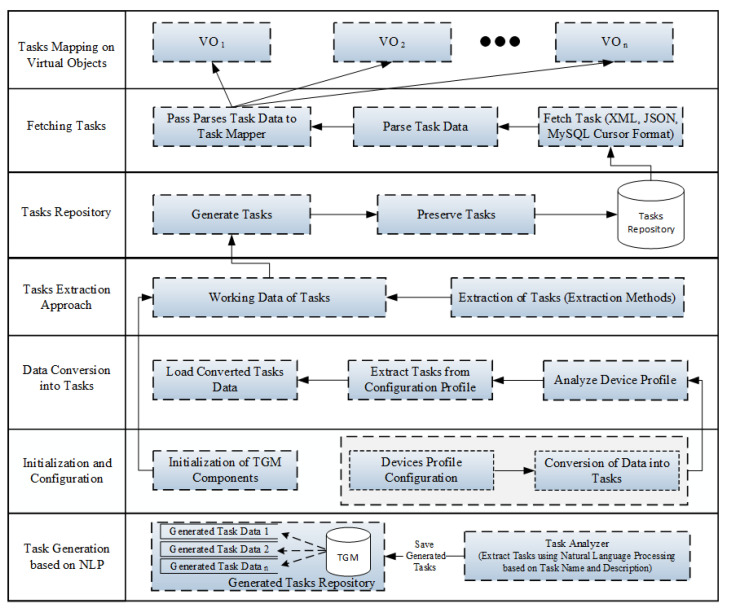
Basic flow of automatic generation of health tasks.

This subsection presents the service analysis and decomposition layer of the proposed task orchestration architecture. In this layer, users use a self-management approach to enters service titles and concise descriptions. This layer is responsible for analyzing and decomposing services into micro-services based on NLP techniques using a service analyzer. The NLP techniques are the most commonly used techniques in text mining to discover hidden insights and useful information from text, such as tokenization, part of speech (POS) tagging, stemming, a bag of words, to name a few. These techniques also help the machine to understand and process the meaning of human languages. The service analyzer uses service title and description as an input and then decomposes the given service into multiple micro-services using NLP techniques. Furthermore, service analyzer uses parts of speech (POS) tagging mechanism to investigate service description to discover a verb, which is essential to identify the type of the micro-service. Finally, the micro-service analyzer is used to examine micro-service to generate input tasks based on a concise description of the micro-service using NLP techniques. The basic flow of the task generator manager (TGC) is illustrated in Figure 3.

Once input tasks are generated, TGC receives generated tasks from the micro-service analyzer and saves them into task data storage. The generated tasks are categorized as periodic or event based on the adverb, followed by the sentence’s main verb. For example, there are the following tasks extracted automatically from the service title and description, such as getting body temperature, getting heart rate, getting patient blood pressure, and getting pulse oximeter. In addition, there are the following tasks to transfer sensing data from sensing devices to doctor using IoT Server, such as report body temperature, report heart rate, report patient blood pressure, report pulse oximeter, etc.

#### 3.3.2. Virtualization of IoT Resources

This layer is responsible for the virtualization of IoT resources, such as the virtualization of physical devices. The virtual resource manager (VRM) creates VOs and provides an interface for performing CURD operations on VOs. These VOs are used to act as physical objects (POs) and hold all the essential properties of the POs along with additional properties of the IoT virtualization environment. The behavior of the VOs is defined based on functions associated with POs and other information, such as URI and location. The VRM is also responsible for identifying communication protocol and other data properties, including data validation. VRM also provides graphical representation to visualize VOs, such as ECG icon graphical represents a heart rate sensor. Finally, we store created VOs through a virtualization mechanism in a virtual object repository.

#### 3.3.3. Task/Virtual Object Pair Generation and Mapping

This layer is used to associate generated tasks with created VOs in order to generate best-matched pairs of input tasks and VOs. The generated tasks are retrieved from the tasks repository, and VOs are fetched from the VOs repository to generate best-matched pairs for the given tasks and VOs for the execution process. Each generated healthcare task is associated with one or more VOs. The combination of VOs and generated tasks are used to form URI. The URI contains information, which is used to measure the output of a certain process. The URI is also used to access physical devices in the proposed task orchestration architecture. The task mapping controller (TMC) is used as a design component to map the input tasks on VOs. It is also responsible for monitoring and managing the overall process of the mapping mechanism. It allows users to use a self-management approach to select a task from the given tasks list and map it on the related virtual object using the drag and drop feature to form a best-matched pair. The drag and drop feature provides a user interface to establish a connection between task and virtual object by dropping a solid line from a task in tasks list to a virtual object in the VOs list. TMC use to visualize the established connection between the tasks list and VOs list. Furthermore, it is used to store a mapping configuration in the task mapping repository for further processing. The typical task mapping entity consists of the following attributes: task id, virtual object id, and created timestamp at which the task mapping occurs. The output of the task mapping process is defined as a message, which contains the following parameters listed in Table 3.

#### 3.3.4. Healthcare Task Scheduling for Efficient Resource Utilization

The main objective of task scheduling is to schedule tasks on IoT devices efficiently and effectively. The task scheduler manager (TSM) is responsible for arranging the correct order of input tasks for efficient execution and resource utilization. TSM uses following task attributes, such as execution time, deadline, priority, urgency, sensing, and control tasks, to plan execution order for the given input tasks. TSM uses three-dimensional space to order tasks execution. The three-dimensional space consists of the following dimensions, such as input task T, virtual object (VO) on which the given task *T* is scheduled, and timestamp *t* at which the task *T* is scheduled on VO. TSM receives task list from task repository, VOs list from VOs repository, and output of TMC to initialize the scheduling process using scheduling algorithm. In this research study, an optimized time-constraint aware scheduling approach is used to plan the execution order of the mapping pairs. It is adequate and robust compared to other scheduling approaches because it executes tasks based on system and user constraints (i.e., idle task time, priorities) and different influential factors, such as current task starvation, missing rate, and delays rate, etc. The proposed optimized time-constraint-aware scheduling mechanism is proposed to minimize task idle time to enhance the performance of the scheduling process. To optimize task idle time, an objective function is developed to enhance the performance of the scheduling mechanism. Furthermore, we used an evolutionary technique, such as particle swarm optimization (PSO), to minimize task idle time based on objective function to provide reliable healthcare services to remote patients. PSO is a widely used population-based optimization technique inspired by the flying behavior of birds swarms in nature, such as swarm (group) of birds searching for food [53]. It is an SP technique based on swarm intelligence and can be adapted in many areas to search for the best possible solution in the given multidimensional space [54]. In PSO, each particle has been evaluated using an objective function (cost function) to determine local best $p_{best}$ and global best $g_{best}$ positions of particles. Then, it uses both $p_{best}$ and $g_{best}$ positions of particles to update the velocities and moved towards an optimal solution. Next, it uses a velocity vector to calculate particle speed and direction. Finally, each particle position has been updated to determine the global optima.

In a smart PHMS, there are two types of healthcare tasks: periodic and event-based healthcare tasks. Periodic healthcare tasks are categorized into two sub-types: priority tasks (PT) and normal tasks (NT). Similarly, event-based healthcare tasks are categorized into two sub-types: urgent event tasks (UET) and normal event tasks (NET). Therefore it is essential to propose an optimized scheduling mechanism to handle emergent healthcare tasks effectively. Hence, our work proposes a PSO-based optimized time-constraint aware scheduling mechanism to dynamically and effectively schedule emergent healthcare tasks. Furthermore, a PSO-assisted optimization model is developed based on objective function to minimize task idle time for enhancing time-constraint aware scheduling algorithm. Table 4 presents summary of the notations used in the optimization problem formulation.

It can be observed that there are several observations, for example, there are *N* number of healthcare sensing devices and *K* number of healthcare tasks for each sensing device. Furthermore, different notations are used in the given formulation, for instance, start time, finish time, execution time, and task idle time, etc. The objective function is defined based on the listed notations to minimize task idle time. Task idle time is defined as the difference between $Finish_{time}$ and $Start_{time}$ as shown in Equation (11).

(11) $$Finish_{time}=Execution_{time}+Start_{time}$$

Thus, task idle time is defined as follows in Equation (12):

(12) $$Task_{idle_{time}}=\left|\sum_{i}^{k}\left(Start_{time}-Finish_{time}\right)\right|$$

Our defined objective function aims to minimize $Task_{idle_{time}}$ than predetermined value. The final objective function is presented in equation to minimize completion time of healthcare task to provide efficient healthcare services to remote patients.

(13) $$Task_{idle_{time}}=Minimize\left(\left|\sum_{i}^{k}\left(Start_{time}-Finish_{time}\right)\right|\right)$$

There are following constraints with objective function to minimize $Task_{idle_{time}}$ for effective scheduling of healthcare tasks.

- $Start_{time}$ must be greater than 0 and less than $Finish_{time}$, For instance, $0<Start_{time}<Finish_{time}$.
- $Execution_{time}$ of healthcare task must be greater than 0 $\left(Execution_{time}>0\right)$.
- 1<=i<=k

Furthermore, two decision measures, such urgency measure (UM) and failure measure (FM), are considered to formulate a decision on whether to execute a task or not. UM defines the priority of tasks at which arrived tasks will be executed. Furthermore, UM is calculated based on NET and periodic PT. It is defined as follows in Equation (14):

(14) $$Urgency_{measure}=Task_{deadline\_time}-Task_{finish\_time}$$

Similarly, Algorithm 1 presents a step by step flow to calculate FM between PTs. FM is calculated to decide execution of the given periodic task.
**Algorithm 1** Calculation of Failure Measure for Scheduling of Periodic Tasks.**  function** CALCULATEFAILUREMEASURE(PeriodicTasks)     $Slack_{time}\leftarrow 0$     **for** i←0,n−1 **do**         $Execution_{time\_status}=PeriodicTasks_{deadline\_time}\left[i\right]-PeriodicTasks_{execution\_time}\left[i\right]$         $Slack_{time}=Slack_{time}+Execution_{time\_status}$     **end for**     **if** $Slack_{time}>0$
 **then** Return 1     **else**
        Return 0
     **end if**  **end function**


It is defined based on periodic tasks to decide whether to execute a given periodic task or not. FM ensures that if the scheduler executes a low priority starved task at a given time slot *i*, it will not affect high-priority periodic task execution. Furthermore, it determines task slack for each given periodic task to ensures the safe execution of a task. Slack time can be calculated as the difference between task deadline time and task execution time. Moreover, if the slack time of the given periodic tasks is greater than 0, then FM will be set to 1; otherwise, it will be set to 0.

Algorithm 2 presents the flow of proposed optimized time-constraint aware scheduling mechanism.

**Figure 4:**
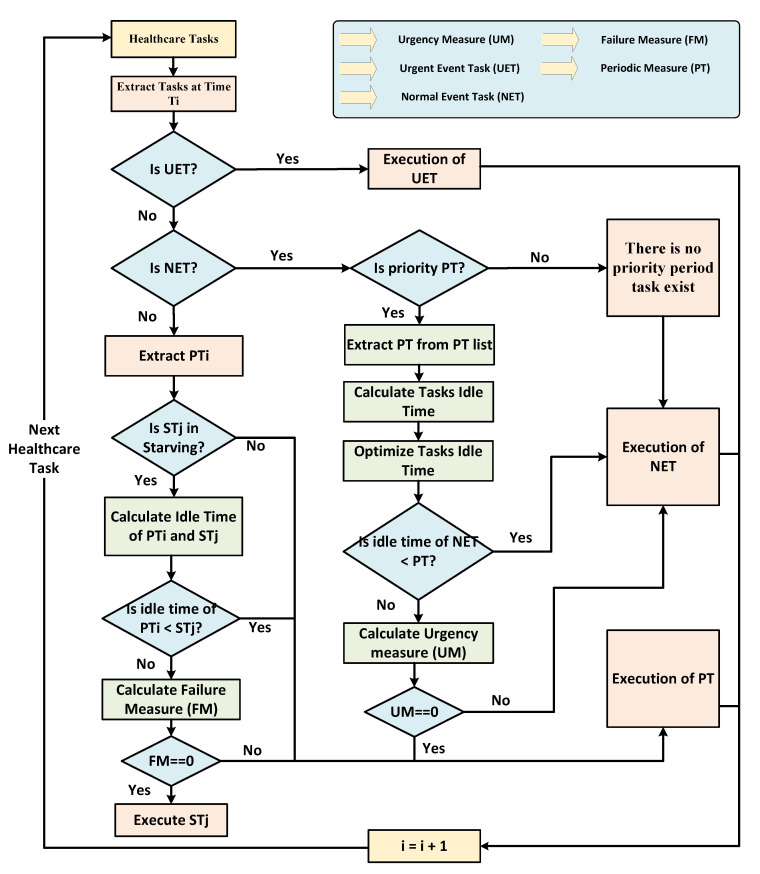
Flow of the proposed time-constraint aware scheduling mechanism.

Furthermore, Figure 4 presents a step-by-step flow of the proposed optimized time-constraint aware scheduling mechanism. In the first step, scheduler fetches healthcare tasks based on arrival times. If an arriving healthcare task is UET, then it executes at the given time interval. If the given healthcare task is NET and there is periodic PT, then task idle time is calculated and passed to the optimization module. PSO-based optimization module receives task idle time and minimizes it based on the objective function. If $O_{NET_{idle\_time}}$ is minimal than $O_{PT_{idle\_time}}$, then schedular executes NET with the nearest deadline; otherwise, UM is calculated to verify that the given time slot can be allocated to a periodic PT or not. If UM is set to 0, then the schedular executes the given periodic PT; otherwise, NET will be executed in the given time slot.

Next, if the given task is periodic PT and there are starved tasks in the starvation tasks list, then extracts starved task (ST) from the starvation list and idle time is calculated for both tasks to be passed to the optimization module aims to minimize task idle time. If the idle time of PT is lower than ST, then the scheduler executes PT at the given time slot; otherwise, FM is calculated to allocate the given slot to execute a starved task or not; otherwise, it executes periodic PT with high priority. FM is calculated using periodic tasks to decide the execution of periodic PT with high priority or execution of starving tasks. The scheduler uses tasks profile and history log data to calculate FM to determine whether to execute periodic PT or starvation tasks with low priority.

The scheduling process re-arranges the execution order of the mapping pairs and adds two additional attributes, such as task execution time and priority, to the existing task mapping process. Once the scheduling process is performed, TSM saves scheduling results in scheduling database in the following format <heathcare_task_id,virtual_object_id,scheduled_ task _time,healthcare_task_priority>.
**Algorithm 2** An Optimized Time-Constraint Aware Scheduling Mechanism**  function** HEALTHCARETASKSSCHEDULING (Tasks[0..n−1], $Task_{i}$)  
▹ Input: Array Tasks[0..n−1] of the given tasks and and $Task_{i}$ is the time step at which tasks arrived in the queue for the execution.      $Urgency_{measure}\leftarrow 0$▹ Urgency measure      $Failure_{measure}\leftarrow 0$▹ Failure measure      **if** $Tasks_{type}=UET$
**then**▹ Urgent Event Task (UET)          Execute the UET      **else if** $Tasks_{type}=NET$
**then**▹ Normal Event Task (NET)          **if** $PT\in Periodic_{task}$
**then**▹ Periodic Task(PT)             Extract PT at time slot *i* from $Periodic_{task}$             Calculate $NET_{idle\_time}$ and $PT_{idle\_time}$ using Equation (12)             $O_{NET_{idle\_time}}=PSO\left(NET_{idle\_time}\right)$             $O_{PT_{idle\_time}}=PSO\left(PT_{idle\_time}\right)$             **if** $O_{NET_{idle\_time}}<O_{PT_{idle\_time}}$ **then**                Execute the NET with nearest deadline             **else**                Calculate $Urgency_{measure}$ using Equation (14)                **if** $Urgency_{measure}=0$ **then**                   Execute the PT with nearest deadline                **else**                   Execute the NET with nearest deadline                **end if**             **end if**          **else**             Execute the NET with nearest deadline          **end if**      **else if** $Tasks_{type}=PT$ **then**          **if** $ST_{i}\in Starvation_{task}$
**then**▹ST represents starved task             Extract ST at time interval *i* from $Starvation_{task}$             Calculate $PT_{idle\_time}$ and $ST_{idle\_time}$ using Equation (12)             $O_{PT_{idle\_time}}=PSO\left(NET_{idle\_time}\right)$             $O_{ST_{idle\_time}}=PSO\left(PT_{idle\_time}\right)$             **if** $O_{PT_{idle\_time}}<O_{ST_{idle\_time}}$ **then**                Execute the High Priority PT             **else**                Calculate $Failure_{measure}$ using Algorithm 1                **if**
$Failure_{measure}=0$ **then**                   Execute Low Priority Starving Task $ST_{i}$                **else**                   Execute the High Priority PT                **end if**             **end if**          **else**             Execute the High Priority PT          **end if**       **else**          Invalid Task Type       **end if**     **end function**


#### 3.3.5. Allocation and Deployment Process of Scheduled Healthcare Tasks

The task allocation manager (TAM) uses scheduling results to allocate the input task on a designated physical IoT device. It is also used to save task/device allocation information. For allocation purposes, TAM uses information from the following repositories, such as task repository, VOs repository, and scheduling repository, to allocate the tasks on the IoT devices; sensors and actuators. Furthermore, TAM uses virtual object ID to track down the status of physical IoT devices in the task allocation process. Finally, a task deployer is used to deploy the task on the corresponding IoT device. It uses to allocate and deploy the tasks based on the order received from the task scheduling manager for allocation purposes. For example, TAM deploys healthcare tasks on corresponding physical devices based on the healthcare tasks scheduling results of the optimized time-constraint aware scheduling mechanism. The URI attribute of the VO is used to access the corresponding physical device.

## 4. Experimental Environment of Proposed Smart PHMS

This section presents the development and experimental environment of the proposed smart PHMS. The proposed smart PHMS uses python as the core programming language for implementing the proposed task orchestration architecture. Python is a high-level, general-purpose programming language. Different researchers and programmers widely use it to develop projects because it is easy to learn, robust and scalable due to frequent version releases [55]. This work uses PyCharm Professional 2020 as an IDE for python programming. Flask framework has been utilized as a web-based lightweight framework to enable customization and accelerate the implementation process. Flask is a lightweight and efficient web-based framework as compared to other frameworks [55,56]. The well-known JavaScript library JSPlumb is used to map tasks on VOs to generate best-matched pairs. Furthermore, MySQL is used as persistence storage, whereas JSON and XML are utilized to fetch data from MySQL to the front-end application. Moreover, Bootstrap has used Bootstrap as a front-end framework to develop a front-end application to achieve dynamic behavior. In Table 5, an implementation environment of the proposed smart PHMS is presented.

**Figure 5:**
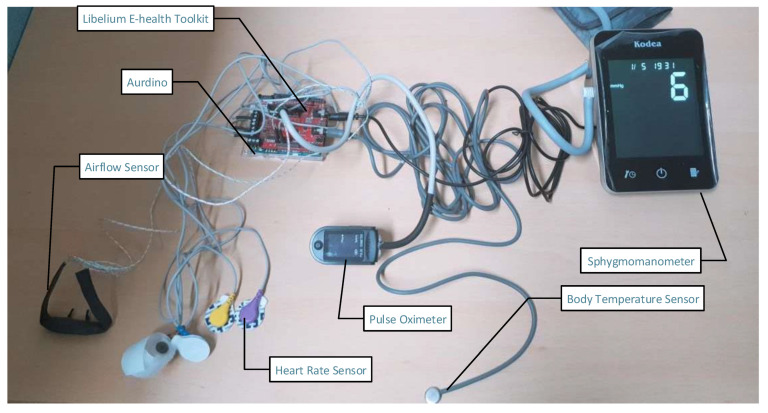
Experimental testbed of proposed smart PHMS.

Figure 5 presents experimental testbed of the proposed smart PHMS. In this work, a Libelium toolkit is used to monitor remote patients health conditions. The e-health toolkit provides nine different sensors for monitoring the real-time health state of remote patients. The information acquired using the e-Health sensor shield can be used to monitor remote patient vital signs or used to acquire sensitive data for analyzing for medical diagnosis. In this work, we have used five different sensors in order to monitor the health conditions of remote patients, such as body temperature, electrocardiogram, pulse oximeter, airflow, and sphygmomanometer sensors. Furthermore, e-Health toolkit provides an open-source API to get sensing data from sensors equipped with remote patients body. In this work, all five healthcare sensors are equipped with remote patients to acquire healthcare data and send sensed data to health service providers, such as hospital, doctors, and nurses. Furthermore, Aurdino is connected with a personal computer (PC) to store sensing data into a database. PC is used to store vital signs data from the Aurdino. Moreover, healthcare sensors are connected with Aurdino to read the vital signs data of patients. The vital signs data of the following sensors like temperature, ECG, blood pressure, pulse oximeter, and airflow are received and processed to monitor patient health. Suppose the reading values of patients and vital signs are abnormal. In that case, event listeners are activated to send alarm notifications in the form of text and voice to the doctor and nurse practitioner to take appropriate action.

Table 6 summarizes the installed healthcare sensors with certain parameters. The certain parameters are normal range, abnormal range, and the main objective of the installed sensors in order to collect valid data from remote patients. The normal range of temperature sensor is from 36.5 °C to 37.5 °C (97.7 °F–99.5 °F), whereas the abnormal range of patient body temperature is from 40.0 °C to 41.5 °C (104–106.7 °F). Similarly, a heart rate sensor is used to measure the electrical activity or rhythm of the heart. The normal range of patient heart rate must be up to 120 ms, whereas the abnormal range must be less than 120 ms. The SpO2 or pulse oximeter sensor monitors the amount of saturation oxygen in the patient’s blood. The normal reading of the pulse oximeter sensor should be between 94% and 100%, whereas if it is less than 90%, then it denotes as abnormal. Similarly, an airflow-based sensor is used to measure the total number of breaths a patient takes per minute. The normal respiration rate should be between 15 bpm and 30 bpm, whereas resting is considered as the abnormal readings. Furthermore, a sphygmomanometer sensor is used to measure blood pressure. It is used to record both systolic and diastolic pressures. The normal range of systolic pressure should be from 90 mm Hg to 119 mm Hg, whereas the normal range of diastolic pressure should be from 60 mm Hg to 79 mm Hg. The abnormal range of systolic pressure is above 119 mm Hg, whereas the abnormal range of diastolic is above 80 mm Hg.

**Figure 6:**
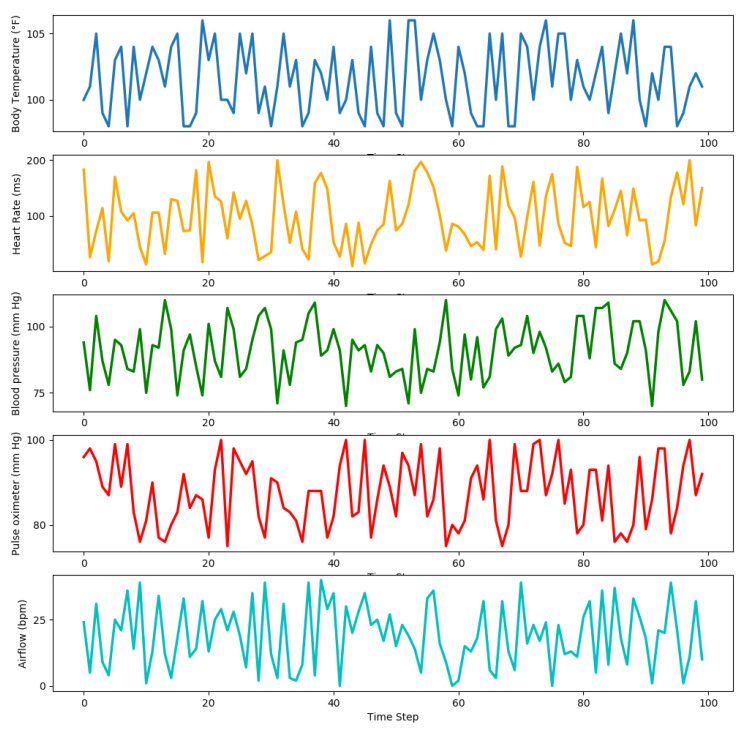
Visualization of healthcare tasks reading data using different sensors.

Furthermore, Figure 6 presents a visualization of vital sign data of remote patients using the Libelium sensors toolkit as discussed earlier in the testbed scenario. In this study, reading of the following vital signs data is visualized, such as temperature, heart rate, blood pressure, pulse oximeter, and respiration rate. It can be observed that the body temperature data varies mostly between the valid temperature ranges (97.8 °F to 106 °F). The normal temperature is 98 °F, and the abnormal temperature is 106 °F. Similarly, It can also be observed that the data of the patient’s heart rate (ECG) varies mostly between the following ranges (0 ms to 200 ms). Normal resting heart rate is 60 to 100 beats per minute (bpm). Abnormal heart rhythms can be described as a heart beating too fast (above 100 bpm) or slow (below 60 bpm). Likewise, reading of patient’s blood pressure data are visualized; it has been analyzed that the blood pressure data varies between 70 mm Hg and 120 mm Hg. The blood pressure data of patients can be classified into the following six categories using systolic and diastolic blood pressures: hypotension, desired, prehypertension, stage 1 hypertension, stage 2 hypertension, and hypertensive crisis. Equation (15) specified the categories of blood pressure using systolic blood pressure $S_{bp}$ and diastolic blood pressure $D_{bp}$ readings.

(15) $$Blood\_Pressure_{Categories}=\left\{\begin{array}{cc} Hypotension, & S_{bp}<90\;and\;D_{bp}<60\\ Desired, & 90\leq S_{bp}\leq 119\;and\;60\leq D_{bp}\leq 79\\ Prehypertension, & 120\leq S_{bp}\leq 139\;and\;80\leq D_{bp}\leq 89\\ Stage\;1\;Hypertension, & 140\leq S_{bp}\leq 159\;and\;90\leq D_{bp}\leq 99\\ Stage\;2\;Hypertension, & 160\leq S_{bp}\leq 179\;and\;100\leq D_{bp}\leq 109\\ Hypertensive\;Crisis, & S_{bp}\geq 180\;and\;D_{bp}\geq 110 \end{array}\right.$$

Furthermore, SpO2 readings are also visualized to analyze pulse and oxygen quantity in patient blood. The normal SpO2 varies between 95% and 100%, as shown below the graph. Normal pulse oximeter readings usually range from 95 to 100 percent. Values under 90 percent are considered low. Abnormal values can be considered as a pulse oximeter is too low (<90). Moreover, readings of the respiration rate of patients are also visualized. The airflow sensor readings show that respiration rate varies between normal and abnormal ranges. For example, the patient’s respiration rate is normal if the reading of the airflow sensor varies between 15 bpm and 30 bpm; otherwise, it will be abnormal. Different vital signs data of patients are received and analyzed to monitor the health status of patients. In case of abnormal readings, our proposed system will alert healthcare practitioners to take appropriate action. In our proposed smart PHMS, event listeners are deployed in the system to inform healthcare practitioners about the emergencies.

## 5. Performance and Comparative Analysis

This section presents performance and comparative analysis results of the proposed smart PHMS.

### 5.1. Performance Analysis

**Figure 7:**
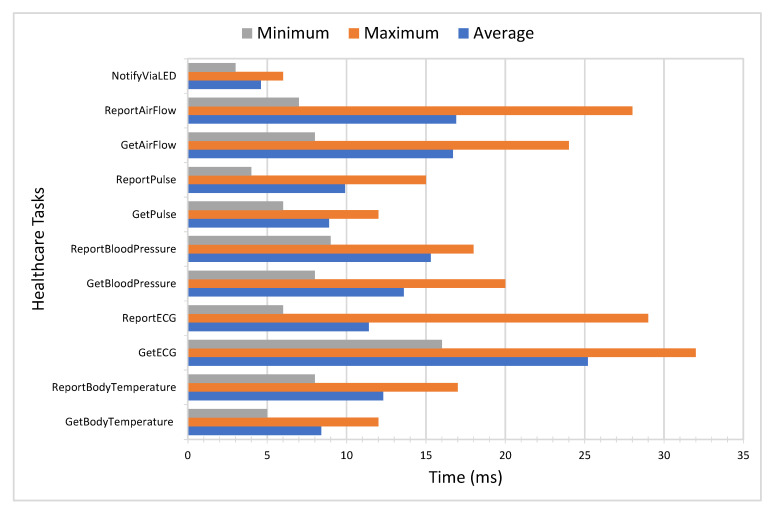
Performance analysis of proposed architecture in terms of round trip time.

This subsection presents RTT, throughput, latency, and response time analysis to evaluate the performance of the proposed smart PHMS. In this research study, a benchmark tool known as Apache JMeter has been utilized to evaluate the performance of the proposed mechanism [58,59,60]. For the simulation of subjects, we used Locust. There are three different subject sets defined, such as 30 subjects, 40 subjects, and 50 subjects, to evaluate the performance of the proposed system. The RTT is defined as the total time takes by the system from the generation of healthcare tasks to the deployment process and response of IoT devices back to the self-management application. Figure 7 is used to present RTT-based statistical analysis, such as minimum RTT, maximum RTT, and average RTT for each given healthcare task. It is evident that the average RTT is 4.6 ms in the case of event-driven healthcare tasks, for instance, ’NotifyViaLED’. On the other hand, the minimum, maximum, and average RTT for the ’GetECG’ task are 16 ms, 32 ms, and 25.2 ms, respectively. Thus, the average RTT is 13.02 for all the executed healthcare tasks, which shows the effectiveness of the proposed smart PHMS. The minimum RTT for the given tasks is 3 ms, and maximum RTT is 32 ms.

**Figure 8:**
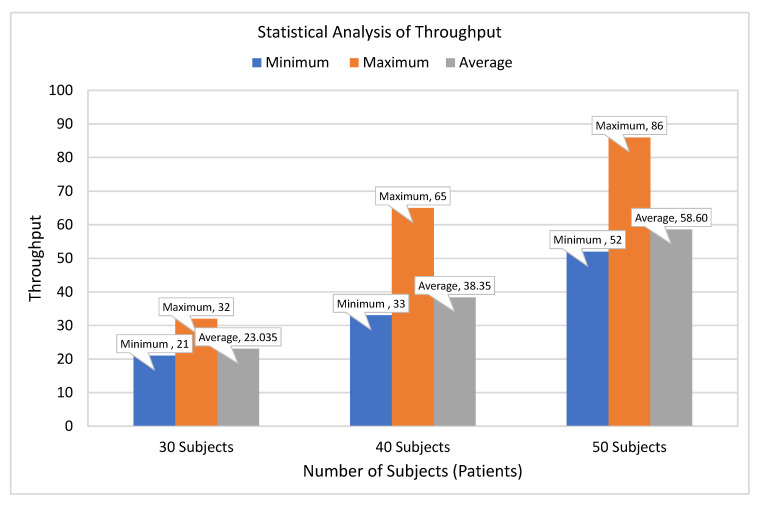
Statistical analysis of healthcare executed tasks per second.

Figure 8 presents different statistical measures to evaluate the throughput of the proposed system using a different set of subjects. The x-axis represents different subjects groups, whereas the y-axis represents throughput as the total number of healthcare tasks executed per second.

It can be observed that minimum, maximum, and average throughput for 30 subjects is 21, 32, and 23 tasks per second. It can also be observed that the throughput of the system improved as the number of subjects increased. It has been analyzed that the throughput of the system gets enhanced as the number of subjects increases from 30 to 40 subjects. Finally, the average throughput for 50 subjects is approximately 59 tasks per second, which shows the usability of the proposed system.

**Figure 9:**
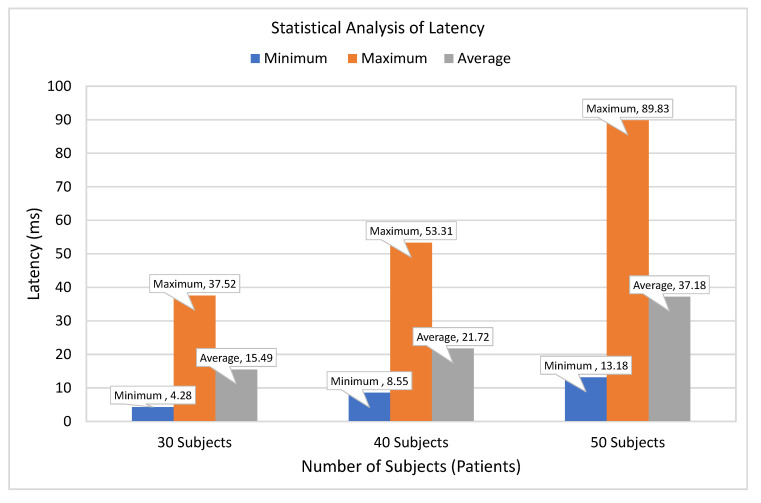
Latency of healthcare tasks deployment.

Figure 9 is used to evaluate the performance of the proposed system in terms of tasks latency. To evaluate tasks latency, we have considered three different subjects to evaluate the latency of executed healthcare tasks in terms of minimum, maximum, and average latency. The average latency for 30 subjects is 15.49 ms, whereas minimum and maximum latency for 30 subjects are 4.28 and 37.52 ms, respectively. Similarly, in the case of 40 subjects, the average latency of executed tasks also get increased from 15.49 ms to 21.72 ms. Furthermore, the average latency of executed tasks for 50 subjects is 37.18 ms. Thus, the statistical analysis shows that the average latency of executed tasks increases slightly as the number of subjects increases, which indicates the significance and reliance of the proposed smart PHMS.

**Figure 10:**
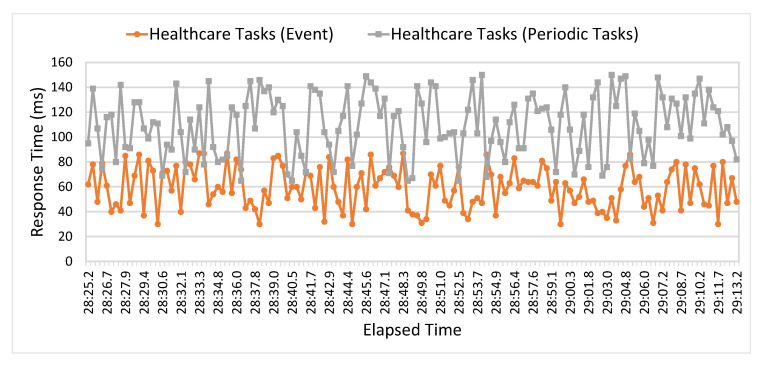
Performance analysis of event and periodic healthcare tasks in terms of Response Time.

In Figure 10, another performance metric is utilized to evaluate the performance of the executed tasks in terms of response time. A comparative analysis is presented to evaluate executed healthcare tasks in terms of average response time. The average response time values of periodic healthcare tasks fluctuate between 62 ms to 150 ms. In contrast, the average response time values of the event-based task are varied between 27 ms to 93 ms. The comparative analysis shows that the average response time of event tasks is slightly low compared to periodic tasks because event-driven tasks have high priority compared to the sensing healthcare tasks.

**Figure 11:**
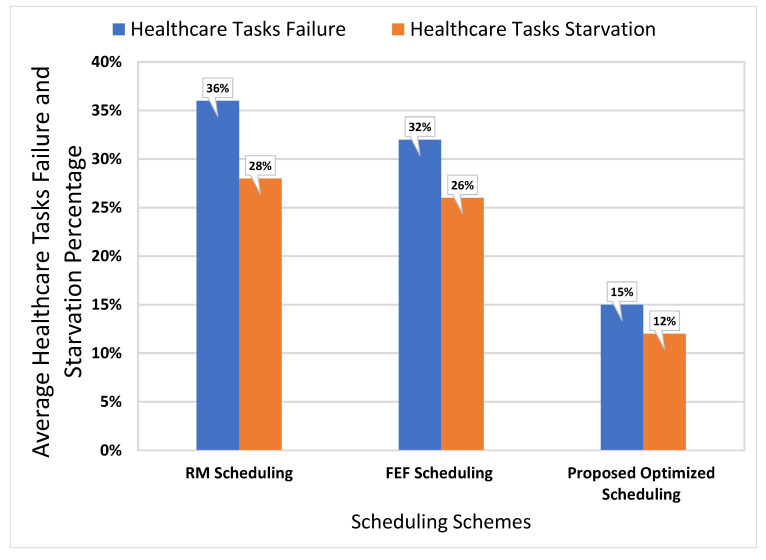
Performance analysis of event and periodic healthcare tasks in terms of Response Time.

In Figure 11, a comparative analysis is presented to compare baseline scheduling mechanisms with an optimized scheduling mechanism to demonstrate the significance of the proposed smart PHMS. The performance analysis of the proposed optimized scheduling mechanism is compared with fair emergency first (FEF) scheduling and rate monotonic (RM) scheduling schemes. The comparative analysis is performed based on the reading of vital signs data of remote patients. The following essential metrics are taken into account to analyze the performance of the scheduling mechanism, such as starvation and drop rate of healthcare tasks.

It is evident that the failure and starvation rate of healthcare tasks using an optimized scheduling mechanism is minimal compared to the FEF and RM scheduling mechanisms. The starvation rate and a dropout rate of our proposed optimized scheduling mechanism are 12% and 15%, respectively. In contrast, starvation and tasks failure rates of the baseline RM scheduling mechanism are 28% and 36%, respectively. Similarly, starvation rate and drop rate of healthcare tasks of the baseline FEF scheduling scheme are 26% and 32%, respectively. Based on the analysis, it is found that the proposed optimized scheduling mechanism reduces tasks starvation rate by 16% and tasks failure rate by 21% compared to the baseline RM scheduling scheme. It can also be analyzed that the proposed scheduling scheme reduces the starvation rate of healthcare tasks by 14% and tasks failure rate by 17%. Thus, the comparative analysis demonstrates that an optimized scheduling mechanism minimizes the starvation and failure rates of healthcare tasks and increases the overall performance of the proposed smart PHMS.

**Figure 12:**
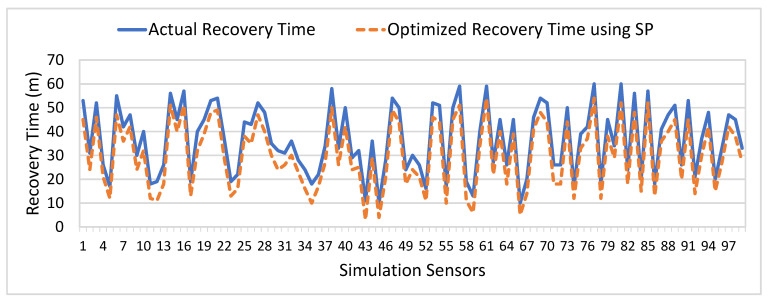
Comparative Analysis of Actual and Optimized Sensors Failure Recovery Time (m).

Figure 12 depicts a comparison of actual recovery time and optimized recovery time of sensors from faults. The y-axis represents the actual and optimized recovery time in which a faulty sensor is replaced with a backup sensor to monitor a patient’s data effectively. The x-axis represents a simulated number of sensing devices used to monitor a patient’s health conditions. The main objective of the optimization module is to minimize the ILR index based on the time taken (in terms of minutes) by sensing devices to recover from the faults. As a result, it can be observed that the recovery time of the faulty sensors is minimized as compared to actual recovery time, which significantly increases the information retrieval ratio and decreases the information lost ratio during the context switching process of the faulty sensor to the backup sensor. The average recovery time of the baseline scheme is 37 min (m), and the average optimized recovery time using stochastic programming (SP) is 30 m. The comparison results proved that our proposed SP approach significantly minimizes the recovery time of the sensors from fault failure and improves the information retrieval ratio for better health data analysis.

**Figure 13:**
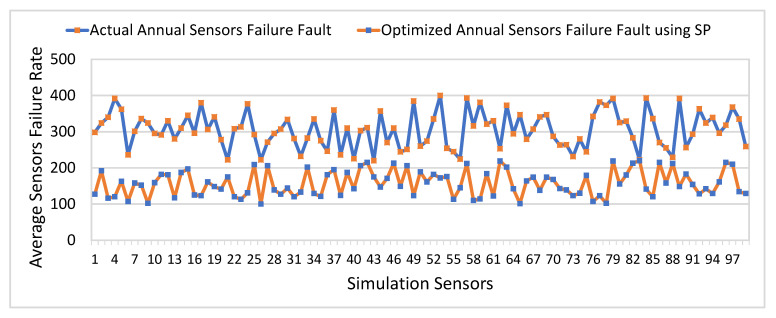
Comparative Analysis of Actual and Optimized Sensors Failure Frequency (Annually).

Similarly, Figure 13 presents a comparison of actual and optimized sensors failure frequency on an annual basis. The y-axis represents the average and optimized sensors failure frequency. The x-axis represents a simulated number of sensors. The main objective of the proposed optimization module is to minimize the sensor’s failure frequency to improves the reliability of the proposed system to provide reliable health services to remote patients. It can be observed that sensors failure frequency is significantly reduced using the optimization scheme compared to the actual annual frequency of sensors failure faults. The average actual frequency of sensors failure is 306, and the average optimized sensors failure frequency is 157 on an annual basis. The comparison results revealed that our proposed SP approach significantly improves the reliability of the proposed PHMS.

### 5.2. Comparative Analysis

This subsection presents a comparative analysis of the proposed smart PHMS with existing IoT-based e-Health systems. To the best of the author’s knowledge, our proposed smart PHMS is the first-ever attempt from the e-Health industry to utilize task orchestration and optimization-based mechanisms to provide reliable and cost-effective health services to patients. There are essential features are considered to demonstrate the effectiveness and significance of the proposed work. These vital features include self-management support, type of application, the simulation of patients data, tasks level management including tasks allocation and tasks scheduling mechanisms, and optimization functionalities to provide reliable and cost-effective health care services. The existing IoT-based e-Health systems, such as H-IOT [8], PHMS [22], HAMS [24], WSN [25], VSM [43], and HI-IOT [61] are developed based on static tasks allocation and tasks scheduling mechanism to monitor patients vital signs data, which cause high latency, low throughput, and high tasks failure. All these existing IoT-based e-Health systems are not well structured to tackle high priority (emergent healthcare tasks) effectively and dynamically. Furthermore, all these existing IoT-based e-Health systems do not support user interfaces for service customization and tasks level management to automatically generate healthcare tasks and schedule tasks to tackle high-priority healthcare tasks effectively. Furthermore, none of the mentioned IoT-based healthcare systems attempted to utilize optimization-based strategies to minimize tasks failure and connection cost to provide reliable health services to patients.

In contrast, our proposed smart PHMS is developed based on tasks orchestration architecture and optimization schemes to monitor patient’s vital signs data, minimize tasks failure, and network connection costs to provide reliable health services. Furthermore, our proposed smart PHMS is well-structured to generate autonomous healthcare tasks based on NLP techniques and a dynamic scheduling approach to handle high-priority health tasks effectively. Moreover, it supports the self-management approach to allow users to interact with a user-friendly interface to customize services related to the healthcare domain. The proposed system is flexible because it supports employing different algorithms for tasks generation, scheduling, and allocation process. Dynamic scheduling, self-management approach, and optimization functionalities make our solution effective and significant in the IoT-based remote healthcare domain. Table 7 presents a detailed analysis of the proposed smart with existing IoT-based healthcare systems.

## 6. Conclusions and Future Work

The provisioning of a cost-effective and reliable health facility is essential for remote patients and is considered the most effective and significant measure for human lives. The vital signs data of patients are used to indicate the status of vital functions of the patient’s body, and thus these vital signs have been used to predict various diseases. The real-time IoT-based systems are an emerging field to support the development of healthcare applications in the IoT context and enable the connection of a vast network of connected devices, which aimed to monitor, process, and analyze patient’s vital signs data to provide better health services. This paper presented a smart PHMS to collect vital signs data of patients in a real-time IoT-based task orchestration architecture using optimized time-constraint-aware scheduling mechanisms. The proposed smart PHMS integrated two core modules: optimized scheduling and optimization of healthcare services modules, using a real-time IoT-based task orchestration to monitor vital signs data of remote patients. An optimized time-constraint scheduling module is developed using a real-time IoT-based task orchestration architecture to generate autonomous healthcare tasks, schedule healthcare tasks in the time domain, and dynamically handle the deployment process for high-priority healthcare tasks. The optimization module is developed using optimization schemes to optimize e-Health services based on objective function to provide cost-effective and reliable services to remote patients. This work utilized the Libelium e-Health toolkit to monitor the vital signs data of remote patients. Additionally, a self-management plane was developed to allow health service providers to customize their services, such as mapping healthcare tasks on corresponding virtual objects. Different performance analysis metrics were utilized to evaluate the significance of the proposed smart PHMS, such as RTT, latency, throughput, response time, and task drop and starvation rates. The experimental results proved that an optimized scheduling mechanism reduces the starvation and failure rate of the healthcare tasks to increase the efficiency of the proposed smart PHMS. The starvation rate of the proposed optimized scheduling mechanism is 12%. In contrast, tasks starvation rate of baseline RM and FEF scheduling mechanism is starvation rate is 28% and 26%, respectively, which indicates that the proposed scheduling mechanism significantly improves the overall performance of healthcare tasks. Similarly, the tasks drop rate of the proposed optimized scheduling mechanism is 15%, which is minimal compared to the RM and FEF scheduling algorithms. Overall, our proposed optimized scheduling mechanism reduced tasks starvation rate by 16% and 14% compared to the RM and FEF scheduling schemes. Furthermore, our optimized scheduling mechanism reduced tasks drop rate by 21% compared to the baseline RM scheduling and 17% compared to the FEF scheduling. The performance analysis reveals that the proposed smart PHMS is an effective and sustainable solution towards a real-time IoT-based e-Health industry to handle the allocation process of emergent healthcare tasks effectively and dynamically. Furthermore, a comparative analysis is performed based on vital factors to demonstrate the effectiveness of the proposed smart PHMS with existing IoT-based e-Health systems. The future direction of the proposed work can be considered to integrate predictive analytics with IoT to predict vital signs data to improve the performance of IoT-based healthcare services. The proposed smart PHMS can also be enhanced by considering more vital signs data to provide reliable healthcare services.

## Figures and Tables

**Table 1:** Summary of the existing healthcare systems.

Application	Platform	Objective	Pros	Cons
Remote health monitoring [22]	IoT	The proposed system was developed to monitor critical events related to cardiac diseases.	Provides real-time alerts, low computation cost to process remote patients data.	No-fault tolerance, static deployment process of healthcare tasks, high cost.
Vital signs monitoring [23]	IoT	This study aimed to record vital signs data of patients, such as ECG, blood pressure, etc.	Correct or reject distorted signals of vital signs based on the data fusion approach.	High energy consumption and static platform for handling emergent conditions
Health monitoring system [24]	IoT	Advanced paradigms of IoT-based system for monitoring critical symptoms related to cardiac disease.	Emergencies alerts, GUI, reduce energy consumption due to Bluetooth.	Delay in emergencies alerts, and low reliability.
F2C model [34]	Cloud-Computing	The main objective is to monitor the health conditions of remote patients based on the fog-to-cloud paradigm.	It uses the F2C model to provide storage and security of healthcare data. It sends emergencies alert using a smartphone.	No-fault tolerance, static scheduling of tasks related to heart rate monitoring.
ECG monitoring system [35]	Cloud-Computing	The authors developed a Cloud-based reliable architecture for ECG monitoring of remote patients in smart health.	High bandwidth rates for healthcare data transmission, and web-based GUI for versatile services.	No scheduling mechanism for handling emergent condition of remote patients.
Heart rate monitoring system [37]	Cloud-Computing	Attempted to monitor heart rate on a continuous basis to facilitate cardiac patients.	Ease of use, GUI, and healthcare data security.	Scalability issues.
BodyCloud [38]	Cloud-Computing	The proposed BodyCloud is a SaaS approach for real-time monitoring cardiac patient’s data.	Scalable approach for healthcare data storage and analysis.	Lack of emergency alerts and global security.
Case study [39]	Fog-Computing	The authors presented a case study to analyze ECG signals to play a vital role in the diagnosis of cardiac diseases.	Real-time monitoring of ECG signals, and bandwidth efficiency.	Lack of emergency alarms and scalability issues.
Smart e-Health [40]	Fog-Computing	Introduction of Fog-layer in IoT-based healthcare platforms for health monitoring in the home and in the hospital.	Reducing latency and improve consistency.	Inefficient consumption of energy, lack of scheduling for handling emergent conditions.
Smart e-Health gateway [41]	Fog-Computing	This study aimed to integrate fog-computing into existing IoT-based healthcare systems for efficient data processing and mining.	Real-time vital signs data processing, and provide mobility support for home and hospital patients.	Interoperability issue for a variety of nodes, lack of dealing with emergent healthcare tasks.
Patient health monitoring [43]	Fog-Computing	The authors proposed patient’s health monitoring system based on fog computing to process and store data at the smart edge gateway.	Real-time health monitoring, data mining, and notification services.	High computational cost at edge node, scalability issue, and static allocation of healthcare tasks.

**Table 2:** Description of notations and symbols used in the formulation.

Notation	Description
$\left\{s_{1},s_{2},\ldots ,s_{n}\right\}$	There are *N* number of sensors (measurements).
$\left\{x_{1},x_{2},\ldots ,x_{n}\right\}$	It indicates collected sensing data for $i^{th}$ sensor at time *t*.
$S_{N}$	It indicates total number of available sensors.
$S_{K}$	It indicates total number of additional sensors.
$I_{R}$	It is total amount of information retrieve from sensing devices.
$I_{L}$	It is total amount of lost information from sensing devices at time *t*.
$R(S_{N},S_{K})$	It represents ratio of available sensing devices and additional (backup) sensing devices.
$R(I_{R},I_{L})$	It represents ratio of retrieve information and lost information.
ILR	It is defined as information loss recovery index.
$T_{1}$	It represents the lower threshold function.
$T_{2}$	It represents the upper threshold function.
a(max)	It is an average value of the maximum value of a sensor measurement signal.
a(min)	It is an average value of the minimum value of a sensor measurement signal.
$R_{q}$	It denotes a quasi-natural ratio.
$S_{f}$	It represents safety factor to minimize sensors failure frequency to increase the reliability of the proposed smart PHMS.
$S_{index}$	It represents safety index based on ILR and $S_{f}$.

**Table 3:** Message profile parameters.

Parameter	Description
Message ID	It is a unique identifier of a message.
Micro-Service ID	It indicates micro-service to which the given task belongs.
Input Task ID	It represents an identifier of the given input task.
Virtual Object ID	It represents an identifier of the given virtual object.
Timestamp	It represents timestamp at which the task mapping process occurred.

**Table 4:** Notations used in optimization problem formulation.

Notation	Description
$\left\{S_{1},S_{2},S_{3},\ldots ,S_{n}\right\}$	There are *N* number of sensing devices.
$\left\{T_{1},T_{2},T_{3},\ldots ,T_{k}\right\}$	There are *K* number of healthcare tasks for each sensing device.
Taskid	It is identifier that represents healthcare task.
$Start_{time}$	It represents start time of the arrived healthcare task.
$Finish_{time}$	It indicates finish time of healthcare task.
$Execution_{time}$	It represents execution time of healthcare task.
$Task_{idle_{time}}$	It is an idle amount of task completion time.

**Table 5:** Development Environment of Proposed Smart PHMS.

System Component	Description
Operating System	Microsoft Windows 10
CPU	Intel ®Core ™ i3-2130 CPU at 3.40 GHz
Primary Memory	PyCharm Professional 2020
Framework	Flask Framework
Libraries	Drools, JSPlumb
Core Programming Language	Python, JavaScript and jQuery
Front End Framework	Bootstrap
Backend Persistence	MySQL
Healthcare Toolkit	Libelium e-Health Sensor Shield V2.0
Hardware	Arduino
Server	CoAP Server

**Table 6:** Threshold for reading vital signs data.

Sensing Devices	Normal Range	Abnormal Range	Objective
Body Temperature Sensor [57]	97.8 °F to 99 °F (36.5 °C to 37.2 °C)	Temperature higher than 100.4 °F (38 °C)	It is used to determine body temperature.
Heart Rate Sensor (ECG) [57]	120≤x≤200	0≤x<120	ECG is used to measure the electrical activity of the heart.
Sphygmomanometer [57]	Systolic: 90<x<120 (mm Hg) Diastolic: 60<x<80 (mm Hg)	Systolic: 90–119 (mm Hg) Diastolic: 60–79 (mm Hg)	It is used to monitor the blood pressure rate. It is used to measure both systolic and diastolic pressures.
Pulse Oximeter (SpO2) [57]	x<120	94−100% (70–100 bpm)	It is used to measure the quantity of oxygen in the blood.
Airflow Sensor [57]	15<x≤31	x<15 or x>31	Airflow sensor is used to measure the respiration rate.

**Table 7:** Comparative analysis of the proposed smart PHMS with existing e-Heath Systems.

E-Health System	Self-Management Support	Type of Application	Real Simulation	Tasks Scheduling	Optimization Functionality	Objective
IoT-SHS [6]	No	Mobile/Web	Yes	No	No	Monitor both patinet health and environmental conditions
H-IoT [8]	Yes	Web-based	No	Yes	No	Vital signs management
PHMS [22]	Partial	Mobile	Yes	No	No	Vital signs management
HMS [24]	No	Mobile	Yes	No	No	Real time monitoring of patient heart condition
WSN [25]	Yes	Web-based	Yes	No	No	Vital signs management
VSM [43]	No	Middleware	No	No	No	Vital signs management and classification of event occurrence
FDS [45]	Yes	Mobile/Web	Yes	No	No	Fall detection system
HAMS [62]	No	Unknown	Yes	No	No	To monitor patient heart pulse and respiration data
HI-IoT [61]	No	Unknown	No	No	No	Monitor patient vital signs data
Proposed smart PHMS	Yes	Mobile/Web	Yes	Dynamic	Yes	Optimal healthcare tasks management, vital signs monitoring, optimization of tasks starvation rate and minimization of tasks failure rate.
